# Barriers and Facilitators to Enrollment and Retention in the National Diabetes Prevention Program: Perspectives of Women and Clinicians Within a Health System

**DOI:** 10.1089/whr.2020.0102

**Published:** 2021-05-11

**Authors:** Katherine Jane Williams Baucom, Mandy L. Pershing, Kaitlyn M. Dwenger, Michelle Karasawa, Jessica N. Cohan, Elissa M. Ozanne

**Affiliations:** ^1^ Department of Psychology, College of Social and Social Behavioral Science, University of Utah, Salt Lake City, Utah, USA.; ^2^ Division of Health System Innovation and Research, Department of Population Health Sciences, School of Medicine, University of Utah Health, Salt Lake City, Utah, USA.; ^3^ Department of Surgery, University of Utah School of Medicine, Salt Lake City, Utah, USA.

**Keywords:** barriers, diabetes prevention, prediabetes, recruitment, retention

## Abstract

***Background:*** More than 10% of US adults are living with type 2 diabetes. The Centers for Disease Control and Prevention established the National Diabetes Prevention Program (National DPP) in 2010 in an effort to delay or prevent this disease among individuals at high risk. Unfortunately, enrollment and retention rates are low. This qualitative study aims to understand barriers and facilitators to enrolling and completing the National DPP among women, and to provide recommendations for improvement.

***Methods:*** Semistructured interviews were conducted with the following: (1) women who were eligible for the National DPP, but declined to enroll (*n*=11); (2) women who enrolled in the National DPP, but did not complete the program (*n*=12); and (3) clinicians who treat women eligible for the National DPP (*n*=12). Transcripts of the interviews were coded using content analysis.

***Results:*** The 35 interviews (23 patients and 12 clinicians) provided further insight into known barriers, such as the cost of the program, the time that it takes, and inconvenient locations. The study also identified previously undiscovered barriers, including the program not meeting participants' expectations and facilitating referrals. Furthermore, improved communication between clinicians, patients, and National DPP staff could ensure that both clinicians and National DPP staff are aware of patients' goals and their individual barriers to success.

***Conclusions:*** Enrollment and retention in the National DPP may be improved with additional communication, more training for National DPP staff to work more closely with participants, adding better incentives to participation, and making the program more accessible through flexibility in time and/or locations.

## Introduction

Diabetes is one of the most common diseases in the United States, with an estimated 13% of the adult populationover 34 million peopleliving with diabetes.^1^ Over 90% of people with diabetes have type 2 diabetes (T2D),^1^ a largely preventable disease.^2^ In addition to those living with T2D, many adults in the United States are at heightened risk for T2D. For example, in 2018, an estimated 88 million US adults had prediabetes, with blood glucose levels above normal, but not yet diagnostic of T2D.^1^

The National Diabetes Prevention Program (National DPP) was developed in 2010 by the Centers for Disease Control and Prevention (CDC) for individuals at heightened risk for T2D, with the goal of slowing disease progression and reducing the incidence of T2D through weight loss.^3^ Participants in the program meet as a group between two and four times a month to work with program facilitators to lose 5%7% of their body weight through eating healthier and being more physically active.^3^ In the original clinical efficacy study of the Diabetes Prevention Program, the lifestyle intervention was associated with a 58% risk reduction in diabetes and an average 12.3-pound weight loss over a follow-up of 2.8 years.^4^ Furthermore, these benefits persisted 10^5^ and 15 years after program completion.^6^ However, enrolling and retaining participants in the National DPP are particularly challenging, with just 10% of participants who begin the National DPP in community or health care settings completing the full year-long program.^7^

Some of the commonly reported factors associated with lack of enrollment in diabetes prevention programs include being younger,^8^ not fluent in English,^8^ program cost,^9,10^ location of the program and transportation,^8,10^ being busy with work,^8^ and a long wait period before enrollment.^11,12^ Some cultural/community barriers to enrollment have also been reported, including poor communication and coordination between the public health sector and providers, lack of knowledge by community members, and poor integration of interests/skills of community members.^13^

Compared with those who stay in diabetes prevention programs, those who enroll, but do not complete programs are more likely to be younger, male, and have a lower income.^14^ Participants who did not complete programs have cited stressors or unstable life circumstances, transportation difficulties, needing childcare, having family obligations, lack of family support, and chronic pain.^11,14^

Just one known study of the National DPP, focused on women of childbearing age, includes in-depth interviews with patients from within a health system.^15^ Given women's higher engagement with the National DPP to date,^16^ there is substantial opportunity for qualitative study in this group. This study extends the research with a qualitative analysis of barriers and facilitators to enrollment and retention within a health system from the perspectives of (1) women referred to the National DPP and (2) referring clinicians. Greater knowledge in this area has the potential to inform future implementation of the National DPP and increase its reach.

## Materials and Methods

### Study design

Semistructured interviews were conducted with 35 participants in the following three groups: (1) women who are eligible for the National DPP, but declined to enroll (*i.e*., nonenrollees; *n*=11); (2) women who enrolled in the National DPP and completed at least one session, but did not complete the 16-session core curriculum of the program delivered over the first 6 months (*i.e*., noncompleters; *n*=12); and (3) clinicians who treat women eligible for the National DPP (*n*=12).

### Participants

Patient participants for this study were women who were overweight and considered to be at high risk for T2D, and thus met CDC National DPP eligibility criteria. The University of Utah (UofU) National DPP program office provided names of individuals who met study inclusion criteria for either the nonenrollee or noncompleter group. The UofU National DPP has been fully recognized by the CDC since 2018 and is offered to patients, employees, and their families, and several community groups. At the time of recruitment for this study, the out-of-pocket cost of the program was $50. Participants in this study needed to be able to converse in English. Women included in the nonenrollee group were offered an opportunity to enroll in the UofU National DPP, but chose not to enroll. Noncompleters were women who enrolled in the UofU National DPP and completed at least one session, but did not complete the core curriculum (*i.e*., did not complete the first 6 months of the year-long program). Recruitment of patients took place from December 2017 to June 2018 with interviews being conducted either at the UofU or over the phone.

Clinicians who were part of the care pathway of women at risk for diabetes were considered eligible for this study. This included primary care physicians and clinicians involved in diabetes prevention efforts, breast cancer prevention, wellness, and/or diabetes. Recruitment of clinicians took place from July 2018 to December 2018 with interviews being conducted in person or over the phone. Informed consent was obtained from all individual participants included in this study.

### Procedures

All study procedures were reviewed and approved by the University of Utah IRB. After women were determined to be eligible for the study by the UofU National DPP, emails were sent to invite participation in the study. If potential participants did not have an email on record, letters were mailed to them. After this initial contact, members of the study team followed up by phone three to five times. Women who enrolled in the study were provided an informed consent cover letter and were scheduled for an in-person or phone interview. After the completion of the interview, participants received a $50 gift card.

Eligible clinicians were those who were in a position to refer patients to the National DPP and were identified through departmental lists and referrals from other clinicians. Potential participants were informed of the study by email and then were contacted by phone for enrollment. If a clinician agreed to participate, they were provided an informed consent cover letter and were scheduled for an in-person or phone interview. Clinicians were not compensated for participating.

### Interviews and data analysis

The semistructured interviews focused on issues related to barriers and facilitators to enrollment and participation in the National DPP. Topics were tailored to the type of participant (nonenrollee, noncompleter, or clinician). The patient topics included were as follows: (1) knowledge about and interest in the National DPP; (2) barriers to enrolling in and/or completing the National DPP; (3) motivating factors for enrolling and/or participating in the National DPP; (4) social or cultural influences; and (5) opportunities for improving enrollment and retention in the National DPP. Clinician interviews focused on five main questions, which were as follows: (1) number/percent of patients seen who are pre-diabetic or at risk for developing diabetes; (2) approach taken with these patients; (3) thoughts about the National DPP and related programs; (4) the motivation for patients to join the National DPP; and (5) opportunities for increasing participation in lifestyle interventions.

Two study team members (E.M.O. and M.K.) conducted the interviews and coded the transcripts using a content analysis method with an inductive approach^17^ in qualitative analysis software.^18^ Interviews were first open coded for meaning units and given headings. These headings were then grouped into categories. Three interview transcripts (one from each group) were reviewed and discussed by the coders to reach consensus. The coding was iteratively updated through periodic discussion between the two coders.

## Results

A total of 35 participants were interviewed (11 nonenrollees, 12 noncompleters, and 12 clinicians). Interviews lasted an average of 31 minutes for patients (range: 2045 minutes) and 26 minutes for clinicians (range: 1560 minutes). Patient participants who did not enroll in the National DPP were more racially/ethnically diverse than those who enrolled in the National DPP, but did not complete the program (Table 1). Of the 12 clinician participants, 8 (75%) were women. The clinicians included family medicine (*n*=7) and internal medicine (*n*=3) physicians, a nurse practitioner, and a wellness specialist. Emergent categories of barriers, facilitators, and opportunities for improvement are presented below, separated by patient and clinician perspectives. Differences discovered based on the two types of patient participants (nonenrollees vs. noncompleters) are described. Representative quotes (Q) are highlighted throughout the results section and detailed in Tables 2^4^.

**Table 1.** Patient Demographics

	Nonenrollees (*n*=11)	Noncompleters (*n*=12)
Age		
Average (SD)	51 (14.0)	52 (14.5)
Racial background		
White (non-Hispanic)	4 (36%)	11 (92%)
Hispanic/White	2 (18%)	0 (0%)
Asian	2 (18%)	0 (0%)
African American	1 (9%)	0 (0%)
Native Hawaiian	0 (0%)	1 (8%)
Unknown	2 (18%)	0 (0%)
Employment status		
UofU employee	2 (18%)	4 (33%)
Non-UofU employee	1 (9%)	8 (67%)
Unknown	8 (73%)	0 (0%)

SD, standard deviation; UofU, University of Utah.

**Table 2.** Barriers: Patient Perspective

Categories	Representative quotes
Barriers to enrolling in program: other health conditions	Q1: Because with the oxygen I just really don't have a whole lot of energyI would if I could.Record 33
	Q2: It was, like I said, a slew of things. The physical shape I was in at the time, as well, and because I was a little bit heavier.Record 6
	Q3: I never talked to them because I really never was diagnosedRecord 92
Barriers to completing program: program not what they expected	Q4: Well, I thought it would be more of here's ideas of meals or here's what a meal should look like, you know what I mean? Instead of us, here, count your calories. Count your fats.Record 13
	Q5: I wanted to learn more. More so I think if they got into like the science of it. It's not more so to scare us, but to remind us, these are the things that can happen if we don't take care of ourselves. Because it's easy to pick up bad habits. You know, it's harder to do the good stuff.Record 20
Barriers to completing program: program not aligned with patient goals	Q6: But you know, whether if you missed, if the goal was four classes and you missed five and you still lost weight, would that mean you weren't successful, I don't think so. So I guess ultimately it would be if your health improved, you know, more than how many classes you went to. Whether that's the A1C goes down, or you lost ten pounds, or even if you didn't lose anything, but you're consistently exercising. So you know, those health benefits, if those had all increased rather than you know, oh you didn't come to enough classes, so you can't have hit success kind of thing.Record 24
	Q7: For me, to maintain weight was really good. I think not gaining weight, maintaining weight, keeping track of what you're doing, meeting those requirements, if they'd emphasize, Oh, you're doing a good job. Don't worry about losing weight because you're not gaining weight and you're eating fairly healthy or at least you're trying to would be helpful in some cases.Record 13
	Q8: For me, being successful in the class would have been lowering that blood sugar number that when I went to the doctor. And I think those would have come from weight loss, but I think weight loss can be so challenging and tricky and such a life long struggle that sometimes, one of the things I'm trying to do is look at being healthier, not necessarily gauging that by the number on the scale. So for that, I mean it feels really good to lose weight, but I also know like are there other measurements like oh, I can hike now. Or this healthy predictor is lower now. Like those things make a difference too.Record 23

**Table 3.** Barriers: Clinician Perspective

Categories	Representative quotes
Barriers of referring program: lack of clinician knowledge about the National DPP	Q1: It's really easy if I'm super confident about location and time I can tell them. I know really well when my dietician is seeing patients and where's she's at and exactly what they'll experience. I have a pretty good idea of what they'll experience in the Diabetes Prevention Program but I don't exactly know where the location is and what the times are. Are they in the evening, in the morning? It's kind of a little bit of a black box.Record 27
	Q2: I think you should kind of more advertise this program because we truly don't know about it. I know about this because I was doing the diabetes project here at the office; why don't you come in and talk to us about this and tell us, This is what it is, blah, blah, blah, this is for your patients. I think you should do more advertising for your program.Record 9
	Q3: I think just feeling knowledgeable about it too, like where are the classes? How much money are they going to cost? Cause I think that's sort of, if the patient starts asking a lot of questions or God forbid they go to the program and they're like, I didn't know it was going to be $500. I feel like that can be another barrier to referring to programs[it's] not that providers don't know how to do it because [all] you have to [do is] type in an order, like refer to DPP, but maybe not knowing exactly what's going to happen after their patients get referred. I have I think probably more knowledge of it than the average PCP, but I think that can be a barrier too sometimes.Record 1
Barriers of referring program: systematic problems or preferences	Q4: Yeah, we don't have access to that [other programs], but I do thinkso Intermountain [Hospital] has this three-pronged approached and it's institutional. And it's like, at least according to what they told me, all our patients get screened and if they have pre-diabetes they are referred. Regardless. They are referred either to Way to Health, which is their DPP, individual nutrition counseling, or Diabetes 101 (which is a one-hour class). Every patient, with pre-diabetes by A1C or glucose tests, gets one of those options. In our system [a different hospital], that is not true. Now is that because we don't have a streamlined system or is that because our providers don't want to be told what to do? I don't know! I think if we had a systematic way to do that [choices for referrals], there would be more referrals.Record 6
Barriers of referring program: referral puts the responsibility on the clinician rather than the patient	Q5: I'm saying, We (the health system) are taking this on and we are going to reach out for you and we are going to help you. And so if the health system drops the ball on that we, then instead of the patient going to be like, Well that didn't work, they come back to me and are like, That didn't work. So when the ball gets dropped it goes back to the primary care doc who referred. And so, whereas with the other stuff you've sort of been like, Here are your choices. Take responsibility and go forward. With the DPP, we're saying we're going to take responsibility for making this work for you and when we (the global we) don't take responsibility for making it work, it goes back to the PCP.Record 4
	Q6: I think, and this is my opinion (I don't know that it's necessarily based in pure science), my opinion is that if you build rapport with the patient and sort of understand what their individual motivations, barriers, etc. are, then that referral can sometimes be better received than just like,you have an abnormal BMI, here's a list of things you need to do. You should go to this program. Like, patients are just going to be like, what? That's probably for me and my current practice the biggest barrier.Record 1
	Q7: I think that if I hand off to DPP, and then they're not able to accommodate but they hand off to something else (whether it's Weight Watchers or something at the Y or something wherever), that's fine; but it feels really hard like if I've gotten them to the point that they're engaged and the answer is, We don't have anything for you, then it's like they're just back in my court.Record 4

National DPP, National Diabetes Prevention Program.

**Table 4.** Opportunities for Improvement

Categories	Representative quotes
Patient perspective	Q1: Well because like I said, everybody is different, it's a different issue with each person in there. It might not be the same, it might be their foot or something, their arm, you know? As an example, something you can more focus on because some people might not have a problem eating this, but they have a problem eating carbs Just kind of ways you can manipulate or change up the program for you, your own individual self.Record 20
	Q2: Usually people have diabetes because their diet isn't healthy, and it has their own habit of the diet, so it's hard to control sometimes. It's clever to have a person to monitor you, and then give you [guidance] Some people encourage you to check your status, it makes [it] easier to get on the right track, so I think that will help.Record 81
	Q3: Like kind of the support of the community that you're with that group. That's always helpful and then just personal responsibility of meeting your goals. But it's also good to have someone check in, because if you are having a hard time meeting your goals, sometimes it's good to have that person that you're accountable to, but also that has recommendation[s], or an idea that maybe you haven't thought of, or seeing that gap in whatever you're doing.Record 96
Clinician perspective	Q4: I think it has to be a multi-pronged approach and I know that's tricky because it's like, oh, I have to approach all these people but I think more and more in primary care we're being encouraged to work in teams.Record 1
	Q5: I think it's kind of multi-people talking to them. So, me talking to them as a provider then having the follow-up phone call or email or snail mail, so just kind ofbecause usually if I talk to them then maybe they listen and maybe not, and then if we have somebody reinforcing this through phone call or mail, so I think just kind of a multiple things.Record 9
	Q6: I'd probably say just continued outreach is probably going to be the biggest thing, whether it be from the programs or just mentioning it from our clinic. And probably, it's like I said, most people when they get a new diagnosis or every couple years we mention, Hey, it would be a good idea to get back into the education program, or just a reminder of things. And most of the time we mention it they'reif they are not able to follow-up right then we kind of leave it at that and it falls in the cracks. So probably just people that are able to outreach and remind patients and talk about the different options and schedules would be the biggest thing.Record 8

### Barriers to enrollment and retention in the National DPP

#### Patient perspective

The location (*n*=4 of 23), cost (*n*=3 of 23), and timing (*n*=15 of 23) of the class were all mentioned as barriers to National DPP enrollment or completion. Location was only described as a barrier by the nonenrollees, and cost was only described as a barrier by noncompleters. However, for two of the noncompleters, the cost ($50 USD) was described as being too low. If the cost was higher, they expressed that they would have had higher expectations for the program and been more motivated to complete the program. One noncompleter mentioned that the cost was too high, but that did not stop her from making it work. The cost of the National DPP was not mentioned by the nonenrollees as a barrier to enrollment. Timing of the National DPP class was described as a barrier by both nonenrollees and noncompleters. Classes conflicted with work schedules, school classes, or other life obligations.

In addition to replicating the above previously identified barriers, nonenrollees identified their current health status (*n*=3 of 11) as another barrier to joining the National DPP. When asked reasons for not joining, patients described various health conditions as barriers to enrollment (Table 2: Q1Q3).

Among noncompleters, a major barrier identified by all was the class not meeting their expectations (*n*=12), including a discrepancy between the information women hoped to learn and the program content (*n*=8), concerns about the class leaders (*n*=4), and dissatisfaction with the class setup (*n*=6). Before coming to the first class, they had an idea of what information they wanted to learn from the class and were disappointed when those topics were not covered in class (Table 2: Q4, Q5).

In addition to expectations they held about the class, some women also felt that their primary individual goals for the program were not consistent with the participant goals the National DPP requires. Out of 19 women who were asked what their definition of success would be or what they thought should be viewed as achieving success in the program, 6 mentioned that attendance would be important and 14 women mentioned that meeting their primary individual goals would be a success for them (*e.g*., developing or maintaining a healthy diet, maintaining weight, lowering A1c levels, or generally increasing physical activity) even if they did not meet the specific goals of the National DPP (Table 2: Q6Q8).

#### Clinician perspective

All clinicians (*n*=12) were asked their thoughts about these programs in general and whether or not they feel there are any barriers to referral, enrollment, or participation for the National DPP or comparable programs. Consistent with patient interviews and results of previous studies, costs of the program (*n*=8), location (*n*=9), and time (*n*=7) were noted as top factors affecting enrollment and retention rates for the National DPP by clinicians. In addition, several clinicians (*n*=3) described their lack of knowledge or a lack of information about the National DPP as a barrier to their referral and women's enrollment in the program (Table 3: Q1Q3). Clinicians (*n*=3) also described the referral and follow-up process within the health system as a potential barrier. For example, clinicians expressed a desire to know the outcomes of internal National DPP referrals they placed, noting this would lead to more referrals (Table 3: Q4). Other system-level processes noted by clinicians as barriers to referral and enrollment included the responsibility being placed on the clinician rather than the patient, the clinician wanting to establish rapport before a referral, or the National DPP not referring patients to other programs when there is no availability (Table 3: Q5Q7).

### Facilitators to enrollment and retention in the National DPP, and opportunities for improvement

All patients were asked their opinion or preferences regarding specific motivators for them to either join and/or stay in the National DPP, and all clinicians were asked to share their thoughts on opportunities for increasing participation in the National DPP. We viewed these as facilitators to enrollment and retention in the National DPP, and opportunities for improvement.

### Patient perspective

All patients (*n*=23) were asked if knowing that weight loss and physical activity (primary goals of the National DPP) could help with the prevention of other diseases would motivate them to participate in the National DPP; only three participants said that knowledge of disease prevention would not make any difference in their participation in the National DPP. Many patients identified diseases that would be most important to prevent besides diabetes, given their own or their family members' experiences with the disease. Heart disease (*n*=7) and hypertension (*n*=6) were the most important to patients to prevent. Patients also mentioned arthritis (*n*=3), cancer (general) (*n*=2), anxiety (*n*=2), depression (*n*=2), heart attack (*n*=2), mental health problems broadly (*n*=1), stroke (*n*=1), asthma (*n*=1), and breast cancer (*n*=1) specifically. Many women reported that preventing all diseases were important (*n*=6), while some reported that specifically breast cancer prevention was important to them (*n*=3).

The National DPP providing additional support from the program leaders (*i.e*., lifestyle coaches) to improve enrollment and retention was positively received (*n*=16). The main perceived benefits were that lifestyle coaches could individualize the program in ways that may not be feasible during class (*n*=6), help participants stay on track and complete the program (*n*=6), and provide additional follow-up in terms of reminders and accountability (*n*=3). Examples of how lifestyle coaches could individualize the program included the follows: helping with tailoring diets and meal planning, facilitating personalized goals, and assisting with life circumstances that may influence their participation in the program (Table 4: Q1Q3). Almost all women (*n*=22) were in support of additional incentives to National DPP participation and completion, such as raffles for gift cards, gym memberships, healthy snacks, or employer incentives (*e.g*., insurance discounts, wellness program points, etc.).

Patients viewed increasing the accessibility of National DPP classes as an opportunity for improvement. They liked the idea of having the National DPP offered closer to work or home locations (*n*=9). Providing the option for online classes (*n*=8) or a hybrid classes with a combination of in-person and online meetings (*n*=12) was liked by patients. Some patients (*n*=8) thought that making the possible times for the program more accessible was an option for improvement, and others (*n*=4) were in support of a lower cost or free option for the program, or it being covered by insurance.

#### Clinician perspective

The 12 clinicians interviewed also identified several factors that could increase enrollment and retention in the National DPP. All clinicians were asked their thoughts on the use of multiple health concerns that are risk motivating to encourage participation in the National DPP or similar programs. Half of the clinicians (*n*=6) were in support of describing the additional health benefits beyond diabetes that result from the National DPP, such as cancer. However, two of these clinicians who expressed that it would be motivating for patients thought it would be hard for patients to make those connections between the diseases. For the clinicians who did not believe it would be helpful to describe risk reduction for other disease as a result of the National DPP (*n*=3), their reasoning was that disease risk is very individual and there is only so much time in a National DPP meeting to talk about additional topics. Two clinicians thought that combining the motivation to prevent other diseases with the National DPP would only be beneficial if targeting certain cultural populations; for example, one clinician expressed that it would not make a difference to combine breast cancer prevention with diabetes prevention unless it was focused on specific groups that had a higher risk of breast cancer, including individuals from Asian, Polynesian, Native American, or Native Alaskan groups. One clinician had no opinion on this topic.

Nine clinicians suggested specific diseases that would be more motivating than diabetes to join a lifestyle change program. All cancers (*n*=4), including breast cancer (*n*=2), were viewed as the most motivating, followed by cardiovascular disease (*n*=3), hypertension (*n*=3), pregnancy complications or infertility (*n*=2), arthritis (*n*=2), mental health (*n*=1), obesity (*n*=1), stroke (*n*=1), and muscular skeletal disease (*n*=1).

Clinicians were asked what opportunities they see for increasing participation in lifestyle interventions. They echoed patient-expressed improvements, with the biggest opportunity being to increase accessibility for the patients in regard to location, time, and cost of the National DPP and similar programs (*n*=5). The creation and use of an incentive program for participants (*n*=2) were also mentioned; however, this was noted as not a sustainable option.

In terms of the enrollment process, clinicians believed increasing care team and/or clinician involvement (*n*=5) would increase enrollment. Incorporating multiple members of a patient's care team in the referral and follow-up process was an important related suggestion (*n*=3) (Table 4: Q4Q6). Another clinician expressed the importance of referring clinicians following up with the patient after the initial referral. Finally, the need for continued follow-up contact with the patient in terms of long-term outreach was expressed as helpful for getting patients to join in lifestyle intervention programs.

## Discussion

Our study has identified barriers that have not yet been addressed in the literature, from the perspective of both clinicians and participants, including issues with the program not meeting participants' expectations and focusing on goals different than those of the patients. Previously identified barriers were also identified in this study; namely, cost of the program,^9,10^ the time that it takes,^8^ and inconvenient locations^8,10^indicating the continued importance of addressing these barriers when implementing these programs.

While patients and clinicians had differing views of barriers, there was some overlap between these two groups in terms of facilitators and recommendations. One major overlap addressed the nature of the National DPP staff. Participants felt that the staff were not involved closely enough with participants' progress and did not accommodate diverse individual goals that might differ from the base National DPP program goals. Many clinicians wanted more communication with the National DPP in terms of making sure that their patients were reaching adequate services when they were referred to the National DPP. Much of the concern stemmed from the fact that clinicians do not tend to know what happens when a patient is referred to the National DPP. Information that was expressed as missing includes the cost, process, or support of the program. In addition, in cases where the patient returns to the clinician for some reason (*i.e*., lack of program availability, no follow-up contact by the National DPP after the referral was made), there is no clear next step. These barriers may prevent referrals to the National DPP. Some clinicians suggested that there needs to be more adequate advertisement about the nature of the program so that they can make more educated referrals for their patients. Finally, unique to our study was the suggestion that improved communication between clinician, patient, and National DPP staff could ensure that both clinicians and the National DPP staff are aware of patients' individual goals as well as their barriers to success.

Several other studies have also examined factors related to enrollment and retention in the National DPP. One of these studies compared population estimates of eligibility to actual enrollment and found that adults who were eligible, but did not enroll in the National DPP during the first 5 years of its nationwide implementation were more likely to be younger or older (relative to middle aged), Hispanic (relative to non-Hispanic), and male (relative to female).^16^ Over this same time frame, individuals who enrolled inbut did not completethe National DPP were more likely to be younger and racial/ethnic minority participants.^19^ Qualitative studies have identified barriers to enrollment and retention, including childcare, transportation, and scheduling,^10,15^ length of the year-long program, and discomfort with the group modality.^10,15,20^ Identified facilitators to enrollment and retention include intrinsic motivation (*e.g*., wanting to meet the National DPP challenges), as well as extrinsic factors such as connection with Lifestyle Coach and other participants.^10,15^

Next, to address the above-mentioned barriers discovered in our study, we suggest some program modifications. Better advertisement for the National DPP would address some known barriers, as well as new, and would address concerns from both patients and clinicians.

Advertisement for clinicians could include more detailed information regarding program function and flow. Advertisement for potential patients could include more information addressing how the program prevents other diseases, in addition to diabetes. Another main barrier was a lack of trust and support from the National DPP staff. These issues suggest that the National DPP needs to provide better training for their staff members to be more successful at developing rapport with patients and better address patients' specific goals that expand from the National DPP base goals. Another modification suggested by participants was better incentives for meetings goals within the program, such as raffles for gift cards, gym memberships, or employer incentives (*e.g*., insurance discounts and wellness program points). Most importantly, accessibility to the program needs to be expanded to have a better online presence. By addressing these modifications, there could be a potential increase in the enrollment and retention in the National DPP.

### Study limitations

The primary limitation to this study is that the participants may not be representative of the broader population of potential clinicians and National DPP participants, as this study was conducted at just one of the many health systems delivering the program. The barriers and facilitators identified may not be representative of barriers and facilitators in other systems. Generalizability of these findings is also limited by the lack of diversity in the study sample. Although we contacted all women patients who were eligible for the study, racial diversity differed greatly, with more racial diversity in the nonenrollee group compared with the noncompleter group (Table 1). Despite these limitations, findings from this study add to the broader literature in this area.

## Conclusions

Although increasing participation in the National DPP has implications for millions of US adults at risk for T2D, there are many known barriers to participating in this program. Our qualitative study identified a number of important and actionable barriers, including additional advertisement, more training for National DPP staff to work more closely with participants, adding better incentives to participation, and making the program more accessible to people through flexibility in time and/or locations to increase enrollment and retention within the National DPP and help prevent disease in many more people across the country.

## Ethical Standards

All procedures performed in studies involving human participants were in accordance with the ethical standards of the institutional and/or national research committee and with the 1964 Helsinki declaration and its later amendments or comparable ethical standards.

## Abbreviations Used

CDC: Centers for Disease Control and Prevention
National DPP: National Diabetes Prevention Program
SD: standard deviation
T2D: type 2 diabetes
UofU: University of Utah
